# Potential of Silver Nanoparticles in Overcoming the Intrinsic Resistance of *Pseudomonas aeruginosa* to Secondary Metabolites from Carnivorous Plants

**DOI:** 10.3390/ijms22094849

**Published:** 2021-05-03

**Authors:** Marta Krychowiak-Maśnicka, Mirosława Krauze-Baranowska, Sylwia Godlewska, Zbigniew Kaczyński, Aleksandra Bielicka-Giełdoń, Natalia Grzegorczyk, Magdalena Narajczyk, Joanna E. Frackowiak, Aleksandra Krolicka

**Affiliations:** 1 Laboratory of Biologically Active Compounds, Intercollegiate Faculty of Biotechnology UG and MUG, University of Gdansk, 80-307 Gdansk, Poland; nataliagrzegorczyk@g.pl; 2 Department of Pharmacognosy with Medicinal Plant Garden, Medical University of Gdansk, 80-416 Gdansk, Poland; miroslawa.krauze-baranowska@gumed.edu.pl (M.K.-B.); sylwia.godlewska@gumed.edu.pl (S.G.); 3 Faculty of Chemistry, University of Gdansk, 80-308 Gdansk, Poland; zbigniew.kaczynski@ug.edu.pl (Z.K.); a.bielicka-gieldon@ug.edu.pl (A.B.-G.); 4 Laboratory of Electron Microscopy, Faculty of Biology, University of Gdansk, 80-308 Gdansk, Poland; magdalena.narajczyk@ug.edu.pl; 5 Department of Pharmaceutical Technology and Biochemistry, Faculty of Chemistry, Gdansk University of Technology, 80-233 Gdansk, Poland; joanna.frackowiak@pg.edu.pl

**Keywords:** antagonism, *Dionaea muscipula*, *Drosera* spp., fractional bactericidal concentration, activity modulation, naphthoquinone, synergy

## Abstract

Carnivorous plants are exemplary natural sources of secondary metabolites with biological activity. However, the therapeutic antimicrobial potential of these compounds is limited due to intrinsic resistance of selected bacterial pathogens, among which *Pseudomonas aeruginosa* represents an extreme example. The objective of the study was to overcome the intrinsic resistance of *P. aeruginosa* by combining silver nanoparticles (AgNPs) with secondary metabolites from selected carnivorous plant species. We employed the broth microdilution method, the checkerboard titration technique and comprehensive phytochemical analyses to define interactions between nanoparticles and active compounds from carnivorous plants. It has been confirmed that *P. aeruginosa* is resistant to a broad range of secondary metabolites from carnivorous plants, i.e., naphthoquinones, flavonoids, phenolic acids (MBC = 512 µg mL^−1^) and only weakly sensitive to their mixtures, i.e., extracts and extracts’ fractions. However, it was shown that the antimicrobial activity of extracts and fractions with a significant level of naphthoquinone (plumbagin) was significantly enhanced by AgNPs. Our studies clearly demonstrated a crucial role of naphthoquinones in AgNPs and extract interaction, as well as depicted the potential of AgNPs to restore the bactericidal activity of naphthoquinones towards *P. aeruginosa*. Our findings indicate the significant potential of nanoparticles to modulate the activity of selected secondary metabolites and revisit their antimicrobial potential towards human pathogenic bacteria.

## 1. Introduction

The medical importance of biologically active compounds of natural origin is strongly supported by the substantial sales contribution of phytomedical products in the drug market [1]. The emergence of bacterial drug resistance, followed by the growing demand for new antimicrobial approaches, has drawn a lot of attention to secondary metabolites from plants [2]. To date, there have been a few hundred reports on the antimicrobial potential of plant extracts and their active constituents as well as their use to combat antibiotic-resistant pathogens [3]. Carnivorous plants comprise a heterophyletic group of plants producing various biologically active compounds [4]. A family of prey-capturing sundews (Droseraceae), i.e., *Dionaea muscipula* and *Drosera* spp., is the exemplary source of compounds with antimicrobial activity [5]. Secondary metabolites found in tissues of this species belong mainly to three groups of plant phenolics: naphthoquinones (e.g., plumbagin, ramentaceone), flavonoids and phenolic acids [6,7]. 

Although extracts from carnivorous plants and their active constituents show strong antifungal activity [8,9] and high antibacterial potential towards gram-positive bacteria, they are less efficient against gram-negative bacteria [10,11,12]. It was shown that an extreme example of a pathogen that is resistant to secondary metabolites from Droseraceae plants is *Pseudomonas aeruginosa* [13,14], a rod-shaped, encapsulated bacterium that leads to highly virulent infections [15]. Its acquired multidrug resistance, as well as intrinsic resistance to variety of antibiotics and biologically active compounds, pose a serious threat to human health and life [16,17]. However, according to our previous report on the synergistic mixtures of carnivorous plant extracts and nanoparticles used to combat *Staphylococcus aureus* [18], the antimicrobial potential of secondary metabolites from plants can be enhanced by silver nanoparticles (AgNPs). AgNPs are particles of metallic silver with widely discussed potential in wound treatment approaches, i.e., in formulations used to prevent or remove infections of the skin or subcutaneous tissue [19]. Nano-sized silver comprises a less toxic alternative to silver salts used to treat infections following skin injuries [20], i.e., silver nitrate and silver sulphadiazine [21]. As their ionic counterparts, AgNPs are highly potent antimicrobial agents with broad range antimicrobial activity towards fungal and bacterial human pathogens [22,23,24].

The aim of the study was to investigate the potential of silver nanoparticles to enhance bactericidal activity of carnivorous plants extracts and their components towards *P. aeruginosa.* We employed a comprehensive phytochemical analysis to determine the active constituents of selected carnivorous plant extracts, as well as verify their significance for the interaction with silver nanoparticles in overcoming the intrinsic resistance of *P. aeruginosa.*

## 2. Results

### 2.1. Antimicrobial Potential of Plant Extracts, Silver Nanoparticles and Their Combinations

**Figure 1:**
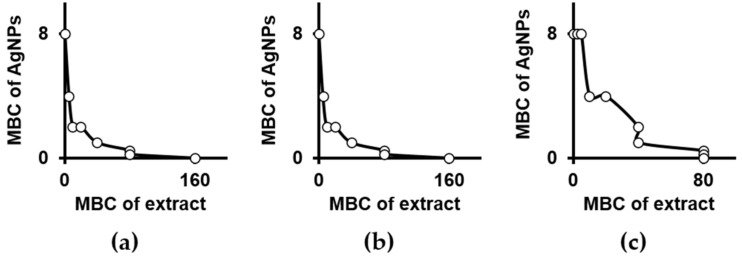
Bactericidal activity of silver nanoparticles combined with extracts from *D. muscipula* (**a**), *Drosera binata* (**b**) and *Drosera gigantea* (**c**) used towards *Pseudomonas aeruginosa* ATCC 27853. Following units apply for axes: µg Ag mL^−1^ (MBC of AgNPs) and µg FW mL^−1^ (MBC of extract). MBC—minimal bactericidal concentration; AgNPs—silver nanoparticles; Ag—silver ions; FW—fresh weight.

Secondary metabolites extracted with tetrahydrofuran from three selected species of carnivorous plants shown limited antibacterial potential against *P. aeruginosa* (Table 1). The MBC of extracts ranged from 80 mg FW mL^−1^ for *Drosera gigantea* to 160 mg FW mL^−1^ for *D. muscipula* and *Drosera binata*. AgNPs, well characterised prior analyses (Figure S1, Supplementary Material), were shown to be highly bactericidal with an MBC equal to 8 µg Ag mL^−1^. In addition, AgNPs applied simultaneously with studied plant extracts enhanced their bactericidal potential and thus reduced effective doses from 50–97% (Figure 1). However, values of FBCI for particular combinations of extracts and AgNPs depicted species-dependent interactions, independent of the antibacterial potential of the extract applied alone (Table 2). Synergy was observed for less active extracts from tissues of *D. muscipula* or *D. binata* combined with AgNPs (FBCI = 0.31), while extracts from *D. gigantea*, with higher bactericidal activity, interacted with nanoparticles only in an additive manner (FBCI = 0.625).

### 2.2. Secondary Metabolites in Plant Extracts

To determine inter-species differences in extract composition, we employed qualitative and quantitative phytochemical analyses. We reported significant differences in the profiles of secondary metabolites between carnivorous plant species used in the study (Figure S2, Supplementary Material). HPLC-DAD-ESI/MS analyses of a key chemical constituent of carnivorous plant tissues, i.e., naphthoquinone plumbagin, revealed that its concentration in extracts corresponds to the synergistic potential of extracts and AgNPs (Table 2). Extracts of both species producing higher levels of plumbagin (over 400 µg mL^−1^), i.e., *D. muscipula* and *D. binata*, were interacting synergistically with nanoparticles, whereas the activity of *D. gigantea* extract (29.2 ± 5.1 µg of plumbagin mL^−1^) was only slightly enhanced (FBCI = 0.625). According to the high synergistic potential and high plumbagin content, *D. muscipula* was selected as a model plant for further experiments.

### 2.3. Identification of Secondary Metabolites from Dionaea muscipula

The presence of flavonoid derivatives of quercetin and kaempferol—three compounds (compound **6**, **8**, **9**), ellagic acid and its mono- and dimethyl derivatives—four compounds (**4**, **5**, **10**, **11**) and naphthoquinones—five compounds (**12**–**16**) was confirmed in the analysed plant material by HPLC-DAD-ESI/MS (Table 3). All of the compounds identified in the extract and fractions from *D. muscipula* were also previously detected in other species of the genus *Drosera* [5,25,26,27]. Among the flavonoid compounds, the presence of hyperoside (**6**) was confirmed by co-chromatography with the standard, while quercetin 3-(6″-*O*-galloyl)-glucoside/or/ galactoside (**8**) and kaempferol 3-(6″-*O*-galloyl)-glucoside (**9**) were identified primarily on the basis of the UV and ESI-MS spectra obtained and their comparison with data from the literature (Table 3) [25,26,27]. Both quercetin 3-(6″-*O*-galloyl)-glucoside and quercetin 3-(6″-*O*-galloyl)-galactoside were earlier identified in *D. muscipula* [27]. It was impossible to determine which sugar moiety forms the structure separated by a quercetin glycoside ester (**8**), because both galloyl-glycoside and galloyl-galactoside gave in ESI-MS spectra the same molecular ion *m/z* values at 617 [M+H] ^+^ and 615 [M-H]^-^, respectively, in positive ion (PI) and negative ion (NI) mode. Similarly, in the group of ellagic acid derivatives, the presence of free ellagic acid (**4**) and 3,3′-*O*-dimethylellagic acid (**11**) was confirmed by comparison with the relevant standards, while 3-*O*-methylellagic acid (**10**) [27] and dimethylellagic acid (**5**) were identified only based on the UV and ESI-MS spectra obtained and compliance of the elution order separated by HPLC mono- and dimethyl- derivatives of ellagic acid with data from the literature [26,28]. The UV spectra obtained for compounds **13**–**16** were similar and had absorption maxima in the range that is characteristic for naphthoquinones [29]. Comparing ESI spectra and t_R_ values with standards among the four compounds mentioned above, two were recognised as plumbagin (**13**) and 3-chloroplumbagin (**14**). Both in the ESI (PI) spectrum of the reference compound-plumbagin and this compound recognised in the analysed samples (**13**) showed double protonated molecules [M+2H]^+^ at *m/z* 190. The presence of such protonated molecules in the ESI-MS spectra of some 1,4-naphthoquinones was described by Chakainresu [30]. On the other hand, in the ESI mass spectra of plumbagin (**13**) and 3-chloroplumbagin (**14**), obtained in negative mode, [M + H]^−^ anions were observed rather than deprotonated molecules, which is due to the reduction of 1,4-naphthoquinones during the electrospray process [31]. In the ESI spectrum of compound **15**, the double protonated molecule [M+2H]^+^ at *m/z* 376 showed that this compound may be 8,8′-biplumbagin [29]. Dihydroplumbagin (**12**) has been identified as a constituent of *D. muscipula* by comparing its HPLC-DAD-ESI/MS parameters, namely: t_R_ (min), UV ƛ_max_ (nm), and *m/z* [M+2H]^+^ with the standard compound (Table 3). To date, only the hydroplumbagin glucoside has been demonstrated in species of the genus *Drosera*, not the free dihydroplumbagin [5,29]. However, dihydroplumbagin was isolated from fresh fruits of *Diospyros maritima* (Ebenaceae) [32]. Dihydroplumbagin and 3-chloroplumbagin have been identified as minor components of the *D. muscipula* analysed. Based on the obtained UV spectra, two compounds **1** and **2** eluted at the lowest t_R_ values (9.4 min and 10.0 min, respectively) were initially recognised as a quercetin derivative (maximum absorption II at 253 nm with a shoulder at 263 nm), while the second was a derivative of kaempferol (II maximum absorption at 265 nm) [33]. The presence of the aforementioned extract constituents was determined in selected fractions, as shown in Table 4.

### 2.4. Antimicrobial Potential of D. muscipula Constituents and Their Interaction with AgNPs

The extraction procedure using tetrahydrofuran with Soxhlet extractor was employed to obtain extracts from *D. muscipula* with the highest possible concentration of chemical constituents. Among the 16 compounds found in the extract, 11 were identified using the HPLC-DAD-ESI/MS technique. A list of secondary metabolites and all data obtained is presented in Table 3. Next, *D. muscipula* extract was partitioned into 6 fractions with a growing gradient of methanol, in order to separate secondary metabolites according to their hydrophobicity and study the role of particular chemicals in synergy with AgNPs. Although SPE fractionation did not allow individual secondary metabolites to be isolated from extracts, it reduced the number of compounds per sample analysed. The presence of extract constituents in fractions was investigated (Table 4) and is summarised in Table 5. Fractions obtained in 0% methanol and 100% methanol were excluded from further analyses, since both were inactive and contained only traces of chemicals (data not shown). Fractions obtained in lower concentration of alcohol (from 20–60%) were dominated by phenolic acids and flavonoids, whereas mostly naphthoquinones were eluted in a higher concentration of methanol (80%). Further antimicrobial analyses revealed that fractions were inactive towards *P. aeruginosa* (MBC > 4.48 g FW mL^−1^) or weakly active with an MBC equal to 4.48 g FW mL^−1^ (Table 5). However, there were significant differences observed between fractions concerning their interaction with silver nanoparticles (Table 5). After the addition of AgNPs, the antimicrobial activity of a fraction obtained in 20% methanol was not affected. Nonetheless, the bactericidal activity of fractions containing both phenolic acids and flavonoids was slightly enhanced to an MBC equal to 2.24 g FW mL^−1^ in the presence of sub-bactericidal doses of AgNPs (i.e., 0.25 × MBC). Interestingly, a synergistic bactericidal effect was observed only when AgNPs were tested simultaneously with the fraction consisting mostly of naphthoquinones. The minimal bactericidal concentration of the fraction was reduced from 4.48 g FW mL^−1^ to 0.14 mg FW mL^−1^; thus, in other words, the lowest bactericidal dose decreased by 97%.

### 2.5. Activity of Selected Secondary Metabolites in Combination with AgNPs

**Figure 2:**
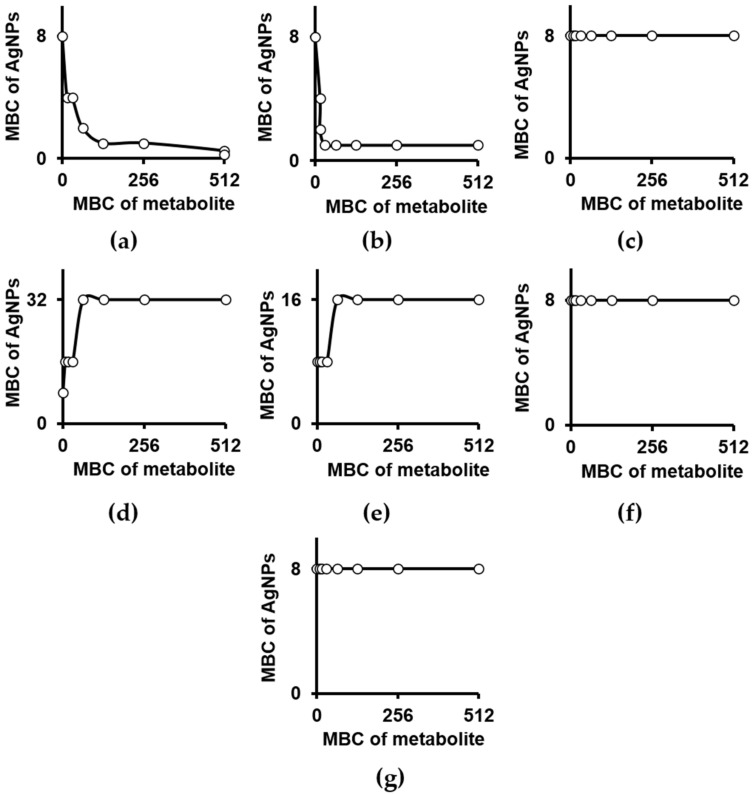
Interaction of silver nanoparticles and secondary metabolites from *D. muscipula* used towards *Pseudomonas aeruginosa* ATCC 27853: (**a**) plumbagin, (**b**) 3-chloroplumbagin, (**c**) quercetin, (**d**) kaempferol, (**e**) ellagic acid, (**f**) 3,3′-di-*O*-methylellagic acid, (**g**) hyperoside. Following units apply for axes: µg Ag mL^−1^ (MBC of AgNPs) and µg mL^−1^ (MBC of metabolite). MBC—minimal bactericidal concentration; AgNPs—silver nanoparticles; Ag—silver ions.

The antibacterial activity of particular pure secondary metabolites found in *D. muscipula* extract was determined, as well as their interaction with AgNPs. Among the identified compounds (Table 3), we studied those which are commercially available, as well as those applied in our previous study [18,34], which represented naphthoquinones (plumbagin, 3-chloroplumbagin), flavonoids and their glycosides (quercetin, kaempferol, hyperoside), as well as phenolic acids (ellagic acid and 3,3′-di-O-methylellagic acid). None of the secondary metabolites studied were active against *P. aeruginosa* in the tested range of concentration, i.e., up to 512 µg mL^−1^. However, the same inactive compounds interacted with AgNPs in distinct manner and we depicted: (i) synergistic interaction for plumbagin (Figure 2a) and 3-chloroplumbagin (Figure 2b), (ii) a lack of interaction for quercetin (Figure 2c), 3,3′-di-O-methylellagic acid (Figure 2f) and hyperoside (Figure 2g), and (iii) an antagonistic interaction for kaempferol (Figure 2d) and ellagic acid (Figure 2e). The fractional bactericidal concentration (FBC) of each compound and FBC indices (FBCI) depicting nanoparticle-compound interactions are summarised in Table 6. According to the synergy with AgNPs (FBCI ≤ 0.5), the activity of naphthquinones was enhanced and the lowest bactericidal concentration of plumbagin and 3-chloroplumbagin in the presence of sub-bactericidal dose of AgNPs (4 µg Ag mL^−1^) was equal to 16 µg mL^−1^ (Table 6, Figure 2a,b). Thus actually, taking into account the pathogen resistance to naphthoquinones, the observed effect comprises an example of the restoration of a compound’s bactericidal activity by silver nanoparticles.

## 3. Discussion

Bacterial infectious diseases represent a serious healthcare issue on a global scale due to the inevitable antibiotic resistance of microorganisms [35,36]. Microbial resistance has a significant medical and economic impact since it limits possible therapies, prolongs hospital stays, and forces the use of expensive third line antibiotics and the development of new drugs [37]. Plant tissues comprise a significant source of secondary metabolites (SMs), i.e., products of secondary metabolism with diverse chemical and biological properties, which are still only partially explored as antimicrobials. Carnivorous plants, especially those from Droseraceae family, were found to produce high levels of various SMs [4]. Although extracts and infusions from these plants were found to be active towards fungal pathogens and Gram-positive bacteria, they are less potent or lack the potential towards Gram-negative bacteria [12,13,14,38,39]. *P. aeruginosa*, i.e., Gram-negative, rod-shaped, encapsulated bacteria which lead to highly virulent infections [15], is an extreme example of a microorganism that is resistant to SMs from carnivorous plants. The scale of its resistance is well depicted by the example of ramentaceone, a naphthoquinone accumulated in tissues of selected *Drosera* sp. According to previous reports, *P. aeruginosa* cells survived at 400 µg mL^−1^ of the naphthoquinone [13], whereas its cytotoxic effect on eukaryotic cell lines is observed at a definitely lower concentration, with an IC_50_ ranging from ca. 0.6 to ca. 9.6 µg mL^−1^ [40]. Our study confirms the weak susceptibility of *P. aeruginosa* to carnivorous plant extracts, previously reported only for *Drosera aliciae* [13,41], as well as its intrinsic resistance to a broad range of secondary metabolites, i.e., naphthoquinones, flavonoids and phenolic acids.

Nonetheless, to further explore and revise the potential of SMs in fighting with resistant *P. aeruginosa*, we employed an approach involving the use of two compounds to enhance the overall biological effect. Such a strategy is widely studied in combination antibiotic therapies as a relevant method with which to overcome antimicrobial resistance [42]. Synergistic interactions of chemicals significantly enhance their biological potential, reduce their effective doses, and delay or prevent resistance development. According to our previous research on *S. aureus*, the antimicrobial potential of extracts from carnivorous plants can be significantly enhanced by silver nanoparticles [18]. Our findings demonstrate that the synergy of nanoparticles and extracts is even more pronounced when their mixture is used towards *P. aeruginosa* (FBC index equal to 0.31) in comparison to *S. aureus* (FBC index equal to 0.53). Thereby, the minimal bactericidal concentration of selected plant extracts and AgNPs used simultaneously can be reduced by 94% and 75%, respectively. Although there are many reports on moderate activity enhancement for drug pairs, such a high level of synergy between antimicrobials is highly specific and not common [43,44,45,46].

The observed synergistic effect of plant extracts and AgNPs was species-specific and referred to the level of plumbagin in plant tissue. Further analyses revealed that among all the other versatile extract components, naphthoquinones, i.e., plumbagin and 3-chloroplumbagin, are the chemicals responsible for the synergy with AgNPs. Interestingly, taking into account the overall resistance of *P. aeruginosa* to naphthoquinones, it can be concluded that the observed synergistic interaction is the example of activity restoration. In the presence of AgNPs, fully resistant bacterial cells were sensitised to compounds, meaning that inactive naphthoquinone became toxic towards bacteria at a significantly lower concentration, i.e., even at 16 µg mL^−1^. A similar efficacy of plumbagin activity enhancement was previously reported for phenylalanine arginine β-naphthylamide (PAβN), an efflux pump inhibitor and outer membrane-permeabilising agent [17]. For the *P. aeruginosa* strain used in our study, we reported the highest reduction of plumbagin bactericidal concentration to 32 µg mL^−1^ by PAβN applied at a concentration equal to 128 µg mL^−1^ [47].

According to the study and our previous reports [18,34], the AgNP‒naphthoquinone interaction is unique and highly specific. However, it is not clear whether its synergistic bactericidal potential against *P. aeruginosa* is based on an interaction with one molecular target or a particular cellular pathway, or comprises the result of multi-target action. The antibacterial activity of both agents relies on complex mechanisms and interactions with many cellular components: silver nanoparticles and ions interact with bacterial cell walls, membranes, proteins and nucleic acids [48], whereas naphthoquinones are alkylating agents inducing oxidative damage in cells [49]. Thus, the determination of AgNP-naphthoquinone synergistic mechanisms is challenging and will have to be further investigated.

Our findings provide new insight into the synergistic antimicrobial approaches based on nanomaterials and plant natural products and clearly depicts the antimicrobial potential and pharmacological relevance of secondary metabolites from carnivorous plants. What is more, synergy among tested agents allows the potential of naphthoquinones to be revised to fight intrinsically resistant pathogens.

## 4. Materials and Methods

### 4.1. Plant Material

Tissues of three carnivorous plants species, i.e., *D. binata*, *D. muscipula* and *D. gigantea*, were obtained under in vitro conditions. Plants were micropropagated on modified half-strength Murashige and Skoog medium [50], supplemented with 2% sucrose, 0.15% charcoal and 0.75% agar (pH 5.5). After a six-month cultivation under white fluorescent light (80 µmol × m^−2^ × s^−1^) at temperatures ranging from 20–22 °C and with a 16 h:8 h (light:dark) photoperiod, plant tissue was collected, washed in distilled water and stored until use at −20 °C.

### 4.2. Bacteria, Antimicrobials and Pure Compounds

*P. aeruginosa* ATCC 27853 was used as a reference strain in all experiments on the antimicrobial potential of the tested agents. The following naturally occurring compounds were purchased from Sigma Aldrich (Saint Louis, MO, USA): plumbagin, kaempferol and ellagic acid. Pure droserone and 3-chloroplumbagin were synthesised by Dr E. Paluszkiewicz (Gdansk University of Technology, Gdansk, Poland), as described earlier [18]. The methylated derivative of ellagic acid, i.e., 3,3′-di-*O*-methylellagic acid, was purchased from Bertin Bioreagent (Montigny le Bretonneux, France).

### 4.3. Silver Nanoparticles

Throughout the study, spherical silver nanostructures coated with mercaptoundecanoic acid (MUA) were used (Prochimia Surfaces Sp. z o.o., Sopot, Poland) as potential modulators of secondary metabolite activity. Metallic core size and shape, hydrodynamic diameter, as well as silver concentrations in nanoparticle formulations were determined by transmission electron microscopy (TEM), dynamic light scattering (DLS) and inductively coupled plasma optical emission spectrometry (ICP-OES), respectively (Supplementary Material).

### 4.4. Plant Tissue Extraction Method

Plant material from *D. binata*, *D. muscipula* and *D. gigantea* was extracted with the ultrasound-assisted method followed by salting-out procedure previously described by Tokarz et al. [51] with modifications. Tetrahydrofuran (THF) was used as the extracting solvent since it penetrates tissues easily and dissolves non-polar compounds. Each 1 g of frozen plant tissue was added to 12 mL of the mixture of THF and water (1:1, *v*/*v*) placed in conical tube. Then, samples were sonicated (35 Hz) for 30 min at room temperature to improve the diffusion of secondary metabolites. After plant tissue was removed from the tubes, 1.5 g of NaCl was added to the remaining liquid and samples were shaken vigorously for 1 min. Next, mixtures were centrifuged (2000× *g*, 5 min) to separate and collect the upper tetrahydrofuran fraction with extracted secondary metabolites. All extracts were stored in a freezer (−20 °C) until used in further experiments. Prior antimicrobial or phytochemical analysis solvent was evaporated from samples and dry residue was dissolved in medium or methanol, respectively. *D. muscipula* tissues were also extracted in Soxhlet extractor with THF as the extracting solvent in order to fractionate a mixture of secondary metabolites.

### 4.5. Fractionation of D. muscipula Extract

The plant tissues used for the fractionation procedure and quantitative phytochemical analyses were extracted in Soxhlet extractor to enhance the efficiency of the extraction procedure. Briefly, 15 g of frozen plant tissue, placed in a Whatman I filter paper tube, was extracted for 12 h with 250 mL of THF heated to its boiling point (66 °C). Then, the extract was collected in THF, evaporated under the stream of air and resuspended to a final concentration of 0.2 g FW mL^−1^, before being stored at −20 °C and used in the fractionation procedure. The solid phase extraction (SPE) technique was used to separate compounds of raw tetrahydrofuran extract from *D. muscipula* according to their hydrophobicity, and thus in further experiments verify their role in synergistic interaction with silver nanoparticles. Fractionation was performed with a vacuum processor (J.T. Baker^®^, Phillipsburg, NJ, USA) on Bakerbond^®^ (J.T. Baker^®^, Phillipsburg, NJ, USA) C18 columns (6 mL, 1000 mg). Each 2 mL of extract was evaporated and resuspended in a minimal volume of pure methanol (100 µL). Next, C18 columns were equilibrated with 6 mL of ultra-pure water, loaded with 100 µL of such prepared extract in methanol and washed with 6 mL of ultra-pure water. To obtain the desired fractions, the following elution gradient of methanol was applied: 0% (6 mL), 20% (6 mL), 40% (6 mL), 60% (6 mL), 80% (6 mL) and 100% (6 mL). Each fraction was collected separately. Samples were then concentrated by methanol evaporation (at 50 °C in a vacuum rotary evaporator) followed by freeze-drying. Dry residue was dissolved in methanol to a final concentration of 0.2 g FW mL^−1^. Methanol was evaporated from samples to dissolve dry residue in culture medium prior to use in antimicrobial assays.

### 4.6. HPLC-DAD-ESI/MS Analyses

The HPLC system (Shimadzu, Tokyo, Japan) consisted of two LC-20AD pumps, a DGU-20A5 degasser, a semi-micro mixer, a CBM-20A controller, CTO-20AC thermostat, SIL 20ACXR autosampler, nitrogen generator (Peak Scientific, Inchinnan, Scotland, UK), and LCMS-2020 mass spectrometer with ESI ionisation. Data were acquired and processed by LabSolution software (version 1.2). The mobile phase consisted of A-water:formic acid (100:0.1, *v*/*v*) and B-acetonitrile:formic acid (100:0.1, *v*/*v*/*v*). The separation was performed on a Kinetex C-18 column (100 mm × 4.6 mm, 2.6 µm) according to the gradient program: 0 min-10% B in A, 50 min-50% B in A, 60 min-100% B in A, 75 min-100% B in A, and 85 min-10% B in A. The column temperature was 20 °C and the flow rate was 1.0 mL min^−1^. The injection volume was 1 µL. UV detection was at λ-254 nm. Mass spectra were acquired by means of the Shimadzu LCMS 2020 in positive (PI) and negative (NI) ion modes. A full-scan (range *m*/*z* 200–800) and SIM (selected ion monitoring) technique for monitoring specific signals was used. The ESI-MS detector parameters were: detector voltage 1.4 kV and 1.6 kV, ionisation potential 4.5 kV and 3.5 kV for PI and NI modes, resp., nebulising gas (N2) flow of 1.5 L min^−1^, desolvation line and block temperatures of 250 and 200 °C, respectively, and drying gas flow (N2) of 15 L min^−1^.

### 4.7. Determination of Antimicrobial Potential

The broth microdilution method was applied to determine bacterial susceptibility to tested agents, as described previously [34]. Experiments were performed according to CLSI guidelines for minimal bactericidal concentration (MBC) determination (CLSI 1996), as follows: a 2-fold serial dilutions of tested agents were performed on 96-well plates containing 100 µL of solution per well. Late logarithmic phase bacterial cultures in cation-adjusted Mueller-Hinton broth (CA-MHB) (Becton Dickinson, Franklin Lakes, NJ, USA) were diluted in fresh medium to a final concentration 1 × 10^8^ CFU mL^−1^ by using McFarland standard as a reference (DensiMeter II, EMO, Brno, Czech Republic). Next, 10 µL of bacterial inoculum with 2.5–5 × 10^4^ CFU was transferred into each well and plates were incubated for 24 h at 37 °C. The content of wells with no visible growth was plated out on Luria agar to determine the number of bacterial cells after incubation (24 h, 37 °C). MBC was defined as the lowest concentration of tested agent that reduces the initial number of bacterial cells by 99.9% after 24 h. The following ranges of concentrations were applied: from 1–128 µg Ag mL^−1^ for AgNPs; from 2.5–320 mg FW mL^−1^ for extracts; from 0.0375–4.48 g FW mL^−1^ for fractions and from 4–512 µg mL^−1^ for pure secondary metabolites.

### 4.8. Determination of Combinatorial Effect of Antimicrobials

The checkerboard titration technique was applied according to the method described earlier [34]. Briefly, concentration gradients of two agents performed by 2-fold dilution on 96-well plate were combined to determine the MBCs of tested agents when applied simultaneously, i.e., the fractional bactericidal concentration (FBC). Next, to assess the nature of interaction of selected pairs of antimicrobials, the FBC index (FBCI) was calculated for their combination according to the equation: FBCI = FBC_A_ + FBC_B_, where FBC_A_ or FBC_B_ are fractional bactericidal concentrations of antimicrobial agent A or antimicrobial agent B. The interaction was defined as synergistic when FBCI was lower or equal to 0.5, additive when FBCI ranged from 0.5–1.0, or antagonistic when FBCI was higher than 1.0. To facilitate the analyses of results for extracts, extract fractions and particular compounds without bactericidal activity towards *P. aeruginosa*, their highest tested concentration (320 mg FW mL^−1^, 4.48 g FW mL^−1^ and 512 µg mL^−1^, respectively) was considered to be equal to or lower than 0.5 × MBC. Results for selected combinations were analysed according to the isobole method [52] and shown as isobolograms.

## Figures and Tables

**Table 1:** Comparison of antimicrobial potential of carnivorous plant extracts.

Extracts	MBC	Unit
*Dionaea muscipula*	160	
*Drosera binata*	160	mg FW mL^−1^
*Drosera gigantea*	80	

MBC—minimal bactericidal concentration. FW—fresh weight of plant tissue.

**Table 2:** Comparison of plumbagin content in tetrahydrofuran extracts from *Dionaea muscipula*, *Drosera binata* and *Drosera gigantea* and their synergistic potential with silver nanoparticles towards *Pseudomonas aeruginosa* ATCC 27853.

Plant Species	FBCI	Plumbagin (µg g^−1^ FW)
*D. muscipula*	0.31	3450 ± 245
*D. binata*	0.31	2035 ± 145
*D. gigantea*	0.625	145 ± 25.5

FBCI—fractional bactericidal concentration index. FW—fresh weight of plant tissue. FBCI ≤ 0.5—synergy; 0.5 ≤ FBCI ≤ 1.0—additive interaction.

**Table 3:** HPLC-DAD-ESI/MS data [t_R_ (min), UV ƛ_max_ (nm), *m*/*z* [M+H]^+^/[M-H]^−^/Ag^+^] of compounds identified in tetrahydrofuran extract from *D. muscipula* obtained in Soxhlet extractor.

Peak Number	t_R_	UV (λ_max_ nm)	MS m/z		Compound [Reference]
			[M+H]^+^/[M-H]^−^/Ag^+^	[M+2H]^+^/[M+H]^−^	
1	9.4	253, 263sh, 302sh, 357	564^+^/-/-	-	unidentified flavonoid, probably quercetin glycoside
2	10.0	265, 298sh, 357	548^+^/-/-	-	unidentified flavonoid, probably kaempferol glycoside
3	10.8	247, 301sh, 350sh, 363	608^+^/-/-	-	unidentified compound
4	11.31	252, 298sh, 366	303^+^/301^−^	-	ellagic acid [25,27]
5	12.00	240, 290sh, 367	331^+^/329^−^/-	-	dimethylellagic acid isomer
6	12.15	253, 266sh, 300sh, 355	465^+^/-/303^+^	-	hyperoside-quercetin 3-*O*-galactoside [5,26,27]
7	13.12	260, 289sh, 350sh	631^−^		unknown compound
8	13.25	258, 266sh, 293sh, 357	617^+^/615^−^	-	quercetin-3-(6′′-*O*-galloyl)-glucoside/galactoside [26,27]
9	15.51	265, 299sh, 351	601^+^/599^−^	-	kaempferol -3-(6′′-*O*-galloyl)-glucoside [25,26]
10	16.90	249, 300sh, 370	317^+^/315^−^	-	3-*O*-methylellagic acid [27]
11	22.75	245, 289sh, 374	331^+^/329^−^	-	3,3′-di-*O*-methylellagic acid [25,27]
12	24.32	267, 346		192^+^/-	dihydroplumbagin [32]
13	31.38	266, 418	-	190^+^/189^−^	plumbagin [5,27]
14	34.17	271, 412		-/223^−^	3-chloroplumbagin [5,27]
15	37.50	276, 414	-	376^+^/-	8,8′-biplumbagin [27]
16	40.90	280, 412	476^+^		unknown naphthoquinone

**Table 4:** Summary of HPLC-DAD-ESI/MS analysis on the presence of compounds in fractionated tetrahydrofuran extract obtained from *D. muscipula* tissues in Soxhlet extractor.

Compound		Presence of Compound in Fraction of *D. muscipula* Extract			
Peak Number	Name	20% MeOH	40% MeOH	60% MeOH	80% MeOH
1	unidentified flavonoid (probably quercetin glycoside)		+		
2	unidentified flavonoid (probably kaempferol glycoside)		+		
3	unidentified compound		++	+	
4	ellagic acid	+	+++	++	+
5	dimethylellagic acid isomer		++	+	
6	hyperoside (quercetin 3-*O*-galactoside)		+	+	
7	unknown compound		+	+	
8	quercetin -3-(6″-*O*-galloyl)-glucoside/galactoside		+	+	
9	kaempferol -3-(6″-*O*-galloyl)-glucoside			+	
10	3-*O*-methylellagic acid		++	+++	+
11	3,3′-di-*O*-methylellagic acid	+		+	+
12	2,3-dihydroplumbagin				+
13	plumbagin		+	+	+++
14	3-chloroplumbagin				+
15	8,8′-biplumbagin				+
16	unknown naphthoquinone				+

Fractions eluted with 0% and 100% of methanol were not included in the table since they contained only traces of extract constituents. + minor compound. ++ major compound. +++ dominant compound.

**Table 5:** Composition and bactericidal potential of fractions from *D. muscipula* tetrahydrofuran extract obtained in methanol gradient in water (20%, 40%, 60% and 80%) combined with silver nanoparticles towards *Pseudomonas aeruginosa* ATCC 27853.

Fraction	Prevalent Secondary Metabolites *	FBC (g FW mL^−1^)	Plumbagin (µg g^−1^ FW)
20% methanol	phenolic acids	>4.48	0
40% methanol	flavonoids, flavonoid glycosides, phenolic acids	2.24	6.14 ± 0.06
60% methanol	flavonoid glycosides, phenolic acids	2.24	5.38 ± 0.02
80% methanol	naphthoquinones, phenolic acids	0.14	1052 ± 12

* according to the results of HPLC-DAD-ESI/MS analyses (Table 3). MBC—minimal bactericidal concentration of agent when tested alone. FBC—fractional bactericidal concentration, i.e., the lowest bactericidal concentration when tested with sub-bactericidal concentration of AgNPs, i.e., 0.25 × MBC (2 µg Ag mL^−1^); FW—fresh weight.

**Table 6:** Antimicrobial potential of selected secondary metabolites from *Dionaea muscipula* in combination with silver nanoparticles towards *Pseudomonas aeruginosa* ATCC 27853.

Secondary Metabolite	FBC (µg mL^−1^)	FBC Index
plumbagin	64	≤0.31
3-chloroplumbagin	16	≤0.128
quercetin	>512	≥1.015
kaempferol	>512	≥2.015
ellagic acid	>512	≥1.015
3,3′-di-*O*-methylellagic acid	>512	≥1.015
hyperoside	>512	≥1.015

MBC—minimal bactericidal concentration of agent when tested alone. FBC—fractional bactericidal concentration, i.e., the lowest bactericidal concentration when tested with sub-bactericidal concentration of AgNPs, i.e., 0.25 × MBC (2 µg Ag mL^−1^). FBC index—fractional bactericidal concentration index.

## Data Availability

All data supporting the conclusions of this manuscript will be made available on request.

## Supplementary Materials

The following are available online at https://www.mdpi.com/article/10.3390/ijms22094849/s1, Figure S1: Characterisation of silver nanoparticles used throughout the study: (a) Transmission electron microscopy (TEM) image of nanoparticles, whereas (b) and (c) represents the size distribution of nanoparticles in water measured with dynamic light scattering (DLS) by intensity and by volume, respectively. Figure S2: Profiles of secondary metabolites in tetrahydrofuran extracts from *Dionaea muscipula* (a), *Drosera binata* (b) and *Drosera gigantea* (c) determined by HPLC/UV-Vis (λ = 254 nm).
