# Supplementary material for: Potential of Silver Nanoparticles in Overcoming the Intrinsic Resistance of Pseudomonas aeruginosa to Secondary Metabolites from Carnivorous Plants
Source: Int J Mol Sci. 2021 May 3;22(9):4849. doi: 10.3390/ijms22094849 (PMC8124972; doi:10.3390/ijms22094849)
Supplement: Supplementary file 1 [file ijms-22-04849-s001.zip › ijms-1166914-supplementary.pdf]

Supplementary material

Potential of silver nanoparticles in overcoming the intrinsic resistance of *Pseudomonas aeruginosa* to secondary metabolites from carnivorous plants

Marta Krychowiak-Maśnicka [1,*], Mirosława Krauze-Baranowska [2], Sylwia Godlewska[2], Zbigniew Kaczyński [3], Aleksandra Bielicka-Giełdoń [3], Natalia Grzegorczyk [1], Magdalena Narajczyk [4], Joanna E. Frackowiak [5] and Aleksandra Krolicka [1,*]

[1] Laboratory of Biologically Active Compounds, Intercollegiate Faculty of Biotechnology UG and MUG, University of Gdansk, 80-307 Gdansk, Poland; nataliagrzegorczyk@g.pl
[2] Department of Pharmacognosy with Medicinal Plant Garden, Medical University of Gdansk, 80-416 Gdansk, Poland; miroslawa.krauze-baranowska@gumed.edu.pl (M.K.-B.); sylwia.godlewska@gumed.edu.pl (S.G.)
[3] Faculty of Chemistry, University of Gdansk, 80-308 Gdansk, Poland; zbigniew.kaczynski@ug.edu.pl (Z.K.); a.bielicka-gieldon@ug.edu.pl (A.B.-G.)
[4] Laboratory of Electron Microscopy, Faculty of Biology, University of Gdansk, 80-308 Gdansk, Poland; magdalena.narajczyk@ug.edu.pl
[5] Department of Pharmaceutical Technology and Biochemistry, Faculty of Chemistry, Gdansk University of Technology, 80-233 Gdansk, Poland; joanna.frackowiak@pg.edu.pl;
* Correspondence: marta.krychowiak@ug.edu.pl (M.K.-M.); aleksandra.krolicka@ug.edu.pl (A.K.)

1. Materials and Methods

1.1. *Silver nanoparticles characterisation*

Inductively Coupled Plasma Optical Emission Spectrometry (ICP-OES) was employed to determine the silver concentration in each of 3 nanoparticle batches and to standardise sample concentrations. The ICP-OES instrument (Perkin Elmer ICP-OES Optima 2000 DV, USA) was optimised before measurements and was used with the following parameters: RF power, 1300 W; frequency 40 MHz, demountable quartz torch, axial viewing, plasma gas (Ar) flow, 15.0 L min^{-1}; auxiliary gas (Ar) flow, 0.2 L min^{-1}; nebuliser gas (Ar) flow, 0.8 L min^{-1}; glass cyclonic spray chamber, sample pump flow rate, 1.5 mL min^{-1}. Ag ions were measured at a wavelength of 328.068 nm. Measurements were performed in 3 replicates for each sample. Transmission Electron Microscopy (TEM) analyses were performed to confirm nanoparticle shapes and sizes. AgNP solutions were applied on copper grids with a 300 mesh (Sigma Aldrich, Saint Louis, MO, USA) coated with a 2% collodion (Sigma Aldrich, Saint Louis, MO, USA) and carbon layer. Particles absorbed onto the surface of grids were examined with Tecnai G2 Spirit BioTWIN electron microscope at 120 kV (FEI Company). Dynamic Light Scattering (DLS) was applied to measure the average hydrodynamic diameter of nanoparticles in water. Briefly, the AgNP solution (15 μL) was added to double distilled H_2O (1.5 mL) placed in polystyrene cuvette and mixed well. Measurements were performed on Zetasizer Nano ZS (Malvern) at 25°C with a He–Ne laser (633 nm, 4 mW), and at a scattering angle of 173°. Smoluchowski approximation, rigorously valid for spherical-like particles, was applied to evaluate results.

1.2. *Qualitative analyses of carnivorous plants metabolites*

High performance liquid chromatography coupled with UV-Vis detection (HPLC/UV-Vis) was employed to preliminarily assess differences in metabolites profile among the tetrahydrofuran extracts from three species, *i.e. Dionaea muscipula*, *Drosera binata* and *Drosera gigantea*. Analyses were performed at room temperature on an Agilent XDB-C18 column (4.6 × 50 mm, 1.8 μm) using a Beckmann Gold System (Beckman Coulter) equipped with a UV-Vis variable wavelength detector Spectra 100 (Thermo

Separations Products) and Rheodyne 7060 injection valve (Rheodyne). Samples of extracts were injected (10 μL) and eluted with the following programme: i) 10–20% A (2 min), ii) 20–40% A (1 min), iii) 40% A (10 min), iv) 40–80% A (5 min), v) 80–90% A (2 min), and vi) 90% A (5 min), where the mobile phase consisted of 0.1% TFA in methanol (A) mixed with 0.1% TFA in water and was used with a flow rate of 1 mL min^{-1}. Extract constituents were detected at 254 nm.

2. Results

2.1. Properties of silver nanoparticles

ICP-OES analyses of AgNPs revealed significant deviations in silver content among three independent batches used throughout the study, *i.e.* the average Ag ion concentration was 5.41 ± 4.93 μg mL^{-1}. Thus, the concentration of AgNPs was standardised according to silver content prior to use in biological assays and each sample was tested in the range of Ag concentrations, *i.e.* from 1 to 128 μg Ag mL^{-1}. However, the granulometric properties of nanoparticles were similar for all tested batches. Nanoparticles were spherical in shape with a metallic core ranging from ca. 1 to 10 nm, as shown in the representative TEM image (Fig. S1a). AgNPs suspended in water formed two subpopulations with the following average hydrodynamic diameter: 160.6 ± 89.52 nm and 16.3 ± 6.959 nm (Fig. S1b). Although subpopulations with bigger particles (160.6 ± 89.52 nm) were present in all tested samples, their contribution to the total volume of the sample was insignificant (Fig. S1c).

2.2. Differences in metabolites' profile among species of carnivorous plants

Qualitative HPLC/UV-Vis analyses revealed significant differences between the three species studied (Fig. S2). The HPLC profiles of extracts from selected carnivorous plants, detected at 254 nm, differed due to the presence of a number of peaks that were not fully separated and of different intensities. We also estimated the retention time of plumbagin (PL), *i.e.* the main naphthoquinone attributed to carnivorous plants (ca. 17 min). Although it was present in all species, the intensity of peaks differed and was significantly lower in extract from *D. gigantea* (Fig. S2c). Further HPLC-DAD-ESI/MS analyses were applied to precisely determine a concentration of PL in all extracts, as well as define active constituents of *D. muscipula*.

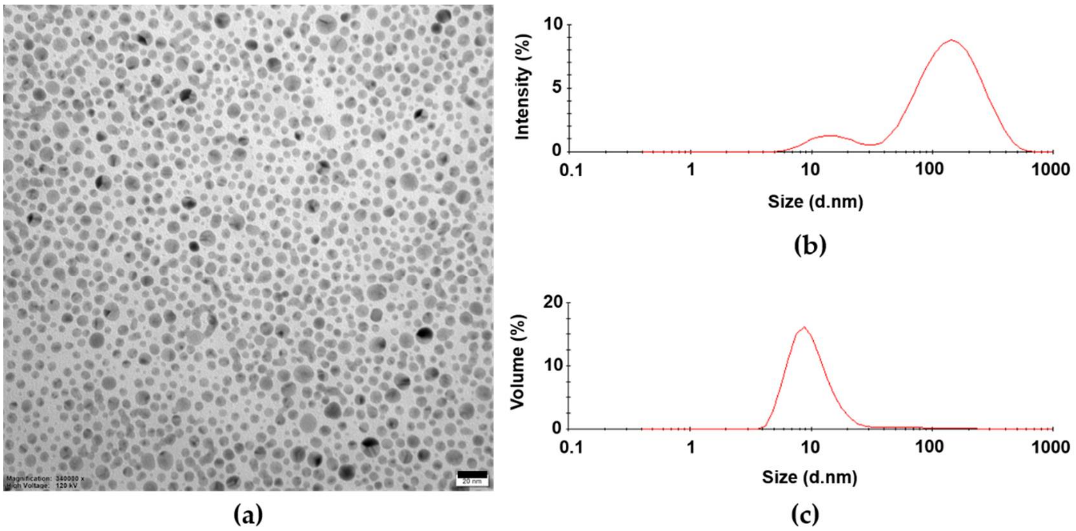

Figure S1. Characterisation of silver nanoparticles used throughout the study: (**a**) Transmission Electron Microscopy (TEM) image of nanoparticles (bar length = 20 nm), whereas (**b**) and (**c**) represents the size distribution of nanoparticles (hydrodynamic diameter) in water measured with Dynamic Light Scattering (DLS) by intensity and by volume, respectively. d.nm., nanoparticles diameter.

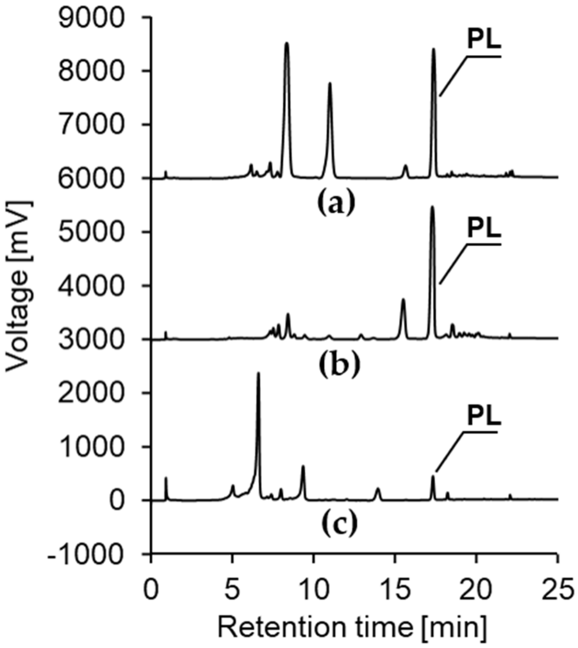

Figure S2. Profiles of secondary metabolites in tetrahydrofuran extracts from *Dionaea muscipula* (**a**), *Drosera binata* (**b**) and *Drosera gigantea* (**c**) determined by HPLC/UV-Vis (λ = 254 nm). PL – plumbagin.
